# Using robot-assisted stiffness perturbations to evoke aftereffects useful to post-stroke gait rehabilitation

**DOI:** 10.3389/frobt.2022.1073746

**Published:** 2023-01-04

**Authors:** Vaughn Chambers, Panagiotis Artemiadis

**Affiliations:** Human-Oriented Robotics and Control Laboratory, University of Delaware, Department of Mechanical Engineering, University of Delaware, Newark, DE, United States

**Keywords:** rehabilitation, robotics, gait, stroke, aftereffects, adaptation, walking, treadmill

## Abstract

Stroke is a major global issue, affecting millions every year. When a stroke occurs, survivors are often left with physical disabilities or difficulties, frequently marked by abnormal gait. Post-stroke gait normally presents as one of or a combination of unilaterally shortened step length, decreased dorsiflexion during swing phase, and decreased walking speed. These factors lead to an increased chance of falling and an overall decrease in quality of life due to a reduced ability to locomote quickly and safely under one’s own power. Many current rehabilitation techniques fail to show lasting results that suggest the potential for producing permanent changes. As technology has advanced, robot-assisted rehabilitation appears to have a distinct advantage, as the precision and repeatability of such an intervention are not matched by conventional human-administered therapy. The possible role in gait rehabilitation of the Variable Stiffness Treadmill (VST), a unique, robotic treadmill, is further investigated in this paper. The VST is a split-belt treadmill that can reduce the vertical stiffness of one of the belts, while the other belt remains rigid. In this work, we show that the repeated unilateral stiffness perturbations created by this device elicit an aftereffect of increased step length that is seen for over 575 gait cycles with healthy subjects after a single 10-min intervention. These long aftereffects are currently unmatched in the literature according to our knowledge. This step length increase is accompanied by kinematics and muscle activity aftereffects that help explain functional changes and have their own independent value when considering the characteristics of post-stroke gait. These results suggest that repeated unilateral stiffness perturbations could possibly be a useful form of post-stroke gait rehabilitation.

## 1 Introduction

On average, every 3 seconds, someone in the world has a stroke. Stroke has been a major concern for decades and only appears to be growing in prevalence, as we’ve seen a 70% increase in stroke cases from 1990 to 2019 (Feigin et al., 2022). A stroke occurs when broken or blocked blood vessels compromise oxygen supply to the brain, which causes death to brain cells. While stroke is indeed an injury to the brain, the death of brain cells can have lasting effects on the whole nervous system and severely impact brain function, speech, and mobility. One of the most common post-stroke issues is gait dysfunction, as an estimated 80% of people lose the ability to walk immediately after having a stroke, and many do not fully regain this ability in the months and years that follow (Duncan et al., 2005). While some disabilities caused by stroke can be fairly common, such as asymmetric gait, it is important to note that stroke is still unique to each individual. Each stroke can affect a different area of the brain, and even a stroke that occurs in the same location has been shown to result in different effects, patient to patient (de Haan et al., 1995; Daly et al., 2010). Because of the prevalence and complexity of stroke, there is much need for robust, patient-specific stroke rehabilitation protocols that allow stroke victims to regain their ability to walk independently and safely.

At a high level, post-stroke gait can usually be characterized by asymmetry. Because stroke often affects just one side of the brain (hemiplegia), one side of the body commonly experiences difficulty in performing motor tasks. Concerning gait, this asymmetry frequently leads to reduced walking speeds (Patterson et al., 2008, 2010), as well as instability and a higher risk of falling (Ugur et al., 2000; Mackintosh et al., 2006). More specifically, post-stroke gait often includes the following behaviors on the affected side: decrease in step length (Titianova et al., 2003), prolonged swing phase (Titianova et al., 2003; Chen et al., 2005; Nadeau, 2014), reduction in overall muscle activity (Olney and Richards, 1996; Chen et al., 2005), prolonged stance phase (Olney and Richards, 1996), less propulsion (Chen et al., 2005), reduced dorsiflexion during swing (Balaban and Tok, 2014), reduced hip and knee flexion during swing (Balaban and Tok, 2014), reduced knee flexion at toe-off (Chen et al., 2005), reduced maximum hip extension (Balaban and Tok, 2014), reduced single support time (Chen et al., 2005), and increased double support time (Nadeau, 2014). For the unaffected leg, common behaviors are: decreased step length (Nadeau, 2014), prolonged stance phase (Olney and Richards, 1996), decreased double support time (Nadeau, 2014), and decreased swing time (Nadeau, 2014).

As stroke is, by definition, an injury to the brain, stroke rehabilitation must consider the brain at some level. One school of thought suggests that to repair the neuronal circuits that are damaged due to cell death during a stroke, repeated and conscious actions are needed to make use of the mechanism of neuroplasticity (Daly and Ruff, 2007; Su et al., 2016). It is believed that through this mechanism, the brain is capable of reorganizing and modifying its structure to allow for better performance and less energy expenditure. The networks in the brain, even throughout adulthood, are not fixed, but instead are always adapting and changing, allowing new tasks to be learned and unused tasks to be forgotten to a degree (Demarin and Morović, 2014). Current theory suggests that for neuroplastic rehabilitation to be most effective, rehabilitation therapy should be repetitive, require focus from the subject, and be similar to the task attempting to be relearned (Daly and Ruff, 2007; Demarin and Morović, 2014; Su et al., 2016).

Robot-assisted post-stroke gait rehabilitation has drawn much interest recently. The inclusion of robotics into the rehabilitation process offers accuracy and repeatability that are not possible with traditional therapy involving clinicians alone (Sale et al., 2012). These robot-assisted strategies have taken on many different forms, ranging from general assistive devices (Peshkin et al., 2005) to body weight supported treadmills (Hesse et al., 1999), to active orthoses (Husemann et al., 2007; Forrester et al., 2011), to full exoskeletons (Nilsson et al., 2014). Overall, these devices have had varying levels of success in terms of post-stroke gait rehabilitation (Hobbs and Artemiadis, 2020).

As discussed above, effective rehabilitation should evoke a neuroplastic response that creates lasting and even permanent changes in a subject’s brain. Since permanent and significant neurological changes are not possible at this time after a single therapy session (Reisman et al., 2009), the main initial indicator of an effective post-stroke rehabilitation protocol is the presence of aftereffects. Aftereffects can be defined as changes in behavior that are evoked by an intervention period and carry over to an unperturbed phase that directly follows the intervention. The behavior that is carried over does not need to be similar to the behavior seen during the intervention; it must only be different from the unperturbed phase before the intervention. These aftereffects first show that during the treatment, the brain is learning and adapting. This leads to changes in a subject’s performance after the treatment has concluded, demonstrating the brain’s ability to make lasting changes with such an intervention. A few studies have shown useful aftereffects toward the goal of post-stroke gait rehabilitation (Reinkensmeyer et al., 2002; Reisman et al., 2009, 2013). One such study (Reisman et al., 2009) produced useful aftereffects with stroke patients using a split-belt treadmill with belts at different speeds. These aftereffects were largely characterized by an increase in step length of up to 5 cm. While this was an impressive result, the aftereffect faded quickly as subjects returned to their baseline behavior after about 25 gait cycles (Reisman et al., 2009). Similar studies using split-belt treadmills have produced similar aftereffects, but have only reported aftereffect durations of a few gait cycles or a few minutes of unperturbed walking (Choi and Bastian, 2007; Huynh et al., 2014). While these studies have used different significance tests, they have all tested for aftereffects using the same general method, comparing post-adaptation gait to baseline gait. Also, these studies tend to focus on the outcome of step length but will occasionally discuss gait cycle timing, kinematics, and muscle activity data. While a useful aftereffect has been achieved in previous studies, much is left to be desired in terms of duration and robustness.

Our lab has developed a novel robotic treadmill that aims to fill the gaps left by previous devices and protocols (Barkan et al., 2014; Skidmore et al., 2015). The Variable Stiffness Treadmill (VST) is a split-belt treadmill capable of varying the vertical stiffness of the interaction between the foot and ground on a single belt (discussed in more detail in Section 2.2). In previous studies, the VST has shown great promise toward becoming an effective post-stroke gait rehabilitation device. The unilateral perturbations created on the VST have displayed the ability to evoke interlimb coordination pathways (Skidmore and Artemiadis, 2015, 2016a,b,c,d, 2019). This coordination between legs is vital to human walking and has been suggested to be controlled at a supraspinal level (Seiterle et al., 2015). Additionally, walking on the VST has been directly shown to elicit significant brain activity responses (Skidmore and Artemiadis, 2016c,d). As discussed above, the brain is the root problem of post-stroke gait dysfunction. Therefore, it is believed that considering the brain is a crucial component of stroke rehabilitation protocol design.

A preliminary experiment was run prior to this study to investigate, for the first time, the aftereffects produced on the VST (Chambers and Artemiadis, 2022). In this study, repeated unilateral stiffness perturbations were used as an intervention with eight healthy subjects. These stiffness perturbations resulted in aftereffects that lasted on average over 200 gait cycles and are meaningful to stroke recovery. These aftereffects were an increase to both left and right step lengths, with the unperturbed side (right) increasing significantly more than the perturbed side (left). While this study was a promising pilot investigation, it had a few shortcomings such as the number of subjects, experiment duration, instrumentation, and depth of analysis.

In this paper, we continue and build upon our previous study (Chambers and Artemiadis, 2022) by performing an in-depth investigation of the aftereffects produced by unilateral stiffness perturbations on the Variable Stiffness Treadmill (VST). We show, with a larger subject pool and a longer experiment length, that repeated perturbations can lead to aftereffects lasting up to 575 gait cycles that appear to possibly have strong implications for post-stroke gait rehabilitation. While the aftereffect of asymmetrically increased step length is further confirmed, other aftereffects regarding kinematics, muscle activity, and ground reaction forces are thoroughly examined. The findings of this paper relate directly to the common issues found in post-stroke gait and suggest that the VST could be an extremely useful tool in advancing the field of post-stroke robot-assisted gait rehabilitation.

## 2 Materials and methods

### 2.1 Overview

**FIGURE 1:**
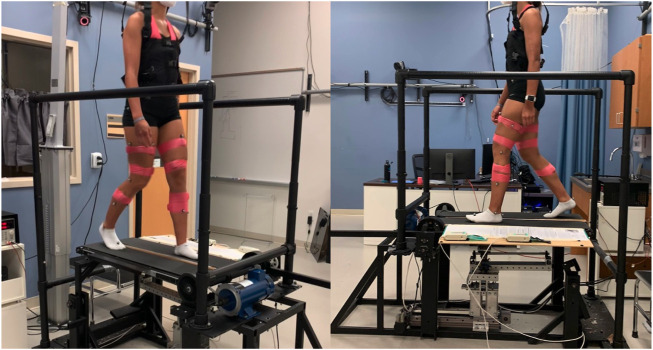
Subject walking on the VST with both belts set to rigid. Reflective markers and EMGs can be seen on the subject, as well as the safety harness.

**FIGURE 2:**
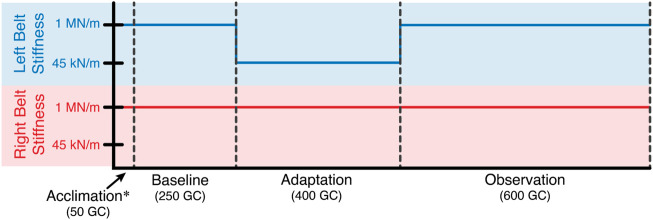
Experiment layout in terms of gait cycles. For the entire experiment, the stiffness of the right treadmill belt remained rigid (1 MN/m). The stiffness of the left treadmill belt was reduced to 45 kN/m for the adaptation phase. Otherwise, the left belt stiffness was also rigid.

Twelve healthy subjects (5 males and 7 females, age: 24 ± 2.98, all right leg dominant) participated in this study. For the entirety of the experiment, subjects walked on the Variable Stiffness Treadmill (see Figure 1). The experiment consisted of 1,300 gait cycles broken into four phases: acclimation, baseline, adaptation, and observation (see Figure 2). During the acclimation phase, subjects walked for 50 gait cycles with both belts of the treadmill set to rigid. The purpose of this portion of the experiment was to allow the subjects to become accustomed to walking on the VST. No data from the acclimation phase was used in the analysis of this study. Next, subjects walked for 250 gait cycles with both sides of the treadmill set to rigid to make up the baseline phase. Unlike the acclimation phase, data from this section of the experiment was used for analysis, as this informed us of each subject’s normal walking behavior. Then, in the adaptation phase, subjects walked for 400 gait cycles with the right treadmill belt stiffness set to rigid and the left treadmill belt stiffness reduced to 45 kN/m. This asymmetric environment caused the subjects to adapt and conform to a new way of walking. Lastly, in the observation phase, subjects walked for 600 gait cycles with both belts set to rigid again, just as they were in the baseline phase. The purpose of this phase was to observe what the subject learned and stored, and what aftereffects carried over to unperturbed walking.

During the entire experiment, subjects were able to select a walking speed that felt closest to their normal pace. All subjects had the options of 90, 95, or 100 cm/s. Additionally, all subjects walked in socks to improve force mat readings during walking (see more details below). While walking, subjects were given three options for what to do with their arms. While the subjects were trying different walking speeds they were asked to swing their arms normally while walking. This would be the ideal posture since it is most like normal walking, but unfortunately, most subjects’ arms block the cameras from seeing the reflective markers on their hips. As an alternative, subjects were asked to either rest the back of their hands on the handrails or gently hold on to the safety harness straps with only their thumb and index finger. These alternative options were given so each subject could walk as comfortably and confidently as possible without offloading much weight or significantly aiding their balance during the low stiffness perturbations. Nine out of 12 subjects chose the method of gently holding onto the harness, one subject was able to swing their arms normally without blocking any markers, and two subjects rested the backs of their hands on the handrails. Little to no variance was observed between these groups of subjects. Additionally, since all analyses and comparisons presented in this study are within each subject (i.e., no comparison between subjects), these slightly different walking postures between subjects were not seen as a major issue. Lastly, subjects were notified verbally of the last 10 gait cycles in each section of the experiment to inform them of stiffness changes. Informed consent was given, while these experimental protocols are approved by the University of Delaware Institutional Review Board (IRB ID#: 1544521-2).

### 2.2 Experimental equipment

The primary device used for this study was the Variable Stiffness Treadmill (VST) (Barkan et al., 2014; Skidmore et al., 2015). This robotic device is a split-belt treadmill, where the belts are tied with respect to speed, but not stiffness. The left belt of the treadmill can reduce its stiffness while the right belt remains rigid. The left belt is capable of stiffness levels ranging from 1 MN/m (which is considered rigid) to about 60 N/m (Skidmore et al., 2015). For this experiment, only two stiffness values were used: 1 MN/m and 45 kN/m. While 1 MN/m feels like walking on a typical treadmill, 45 kN/m is comparable to sand or a soft gym mat. The stiffness level of 45 kN/m was selected after performing multiple pilot studies which tested stiffness levels varying from 20 kN/m to 90 kN/m. Stiffness values much lower than 45 kN/m resulted in significant fatigue from the subjects that was visually identifiable. This fatigue seemed to introduce randomness into the data as gait was strenuous and inconsistent. Stiffness levels much higher than 45 kN/m quickly approached a surface that was too similar to the rigid surface of 1 MN/m. This failed to produce substantial differences between sections of the experiment, and the results were often not statistically significant.

**FIGURE 3:**
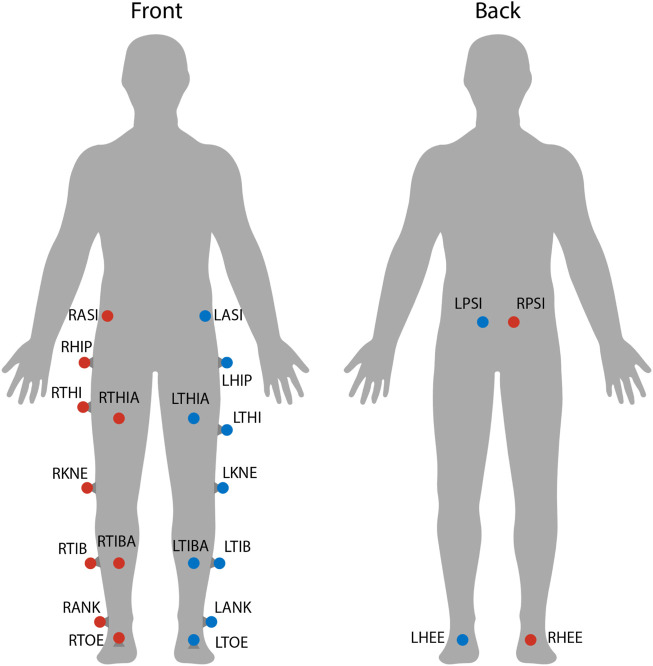
Marker locations on each subject. Twenty-two reflective markers were used for motion capture analysis. The center of mass was estimated as the average between LASI, RASI, LPSI, and RSPI markers.

Each subject’s position in space and its kinematics were collected using a VICON motion capture system. This system includes 8 cameras spaced around the treadmill, each providing data at 100 Hz. Also, 22 reflective markers were placed on each subject’s lower body to allow a lower body skeleton to be produced in VICON Nexus, the software used for labeling markers and processing marker data (see Figure 3). For this skeleton to be created, the following subject metrics are also required: height, weight, leg length, knee width, and ankle width. Raw marker position data and subject metrics are then used to calculate joint angles at the hips, knees, and ankles using VICON Nexus.

Muscle activity was measured during this experiment with 10 surface electromyographic (EMG) sensors (Trigno, Delsys Inc.). Five EMGs were placed on each leg on the following muscles: tibialis anterior (TA), gastrocnemius (GAS), vastus medialis (VA), rectus femoris (RF), and biceps femoris long head (BF). These five muscles were selected as they help explain movement in all three joints of interest (hip, knee, and ankle) in both directions of flexion and extension. Each subject’s skin was prepared by shaving the area (if necessary) and cleaning the area with alcohol wipes. EMGs were attached with double-sided tape and further secured with pre-wrap athletic tape to reduce motion artifact. EMG data were synchronized with motion capture data using a trigger signal from VICON Nexus.

Electromyographic (EMG) data were processed using the following method. For each subject and muscle, the raw data sampled at 2000 Hz was first filtered with a fourth-order Butterworth band-pass filter. Low and high cut-off frequencies of 30 and 300 Hz, respectively, were used. Data were then full-wave rectified. Next, the envelope of the muscle activity data was found by computing a moving average with a window size of 200 data points. Then, a lowpass filter (fourth order, 5 Hz) was used to filter the data again (Shiavi et al., 1998; Singh et al., 2019). The data were then normalized using the maximum value found throughout the experiment. Finally, the EMG data were downsampled to 100 Hz using linear interpolation to match the frequency of the motion capture data. This process produced useful muscle activity data scaled at 0%–100% activity level.

Force mats (Tekscan 3,510 Medical Sensors) were used to collect ground reaction force (GRF) data for the left foot. These mats collect vertical force data in 2068 locations (grid) along the walking surface at 100 Hz. From this data, the total force value and center of pressure location were able to be calculated. Additionally, since the GRF position is needed to create a constant, low-stiffness environment, the real-time center of pressure data were used to update the position of the variable stiffness mechanism on the VST. Through preliminary testing of the force mats, it became obvious that different styles of shoes produce significantly different data, even with the same subject. Because of this, subjects walked in socks to improve force mat readings. Force mat data were also synchronized with motion capture and EMG data using a trigger signal from VICON Nexus. Note that GRF data is only available for the left foot as the VST is only equipped with force sensors on the left belt. While the left GRFs were the main interest to us, future experiments will most likely have GRF data for both feet.

Finally, the subjects were wearing a body weight support harness throughout the experiment, but it was only used as a safety precaution. The harness straps were left with a small amount of slack so that none of the subjects’ weight was offloaded as this could alter their kinematics and GRF data. The harness was worn by each subject around their torso and did not impede walking in any way (see Figure 1).

### 2.3 Data processing

Each subject’s data set, which included marker trajectories, kinematics, muscle activity, and ground reaction forces, was then broken into gait cycles starting at each left heel strike. In other words, a gait cycle was defined from one left heel strike to the next. Heel strike for both legs was detected using a robust kinematic algorithm (Karakasis and Artemiadis, 2021). Then, for each subject, outlier gait cycles were detected using a systematic method that analyzed kinematic data in all three directions at the hip, knee, and ankle, as well as muscle activity data for all 10 muscles (Hobbs and Artemiadis, 2022). Additionally, the last 10 gait cycles of the baseline and adaptation phases were automatically declared as “outliers.” This was because the subjects were verbally informed 10 gait cycles before the stiffness of the treadmill was changed. The experiment was designed in this fashion to ensure that subjects were not surprised, but this given information created an anticipatory effect that was not originally desired. For this reason, this section of data was removed. As stated above, the acclimation phase was not involved in any data analysis and will not be discussed any further.

Data were statistically tested to determine significance. The Wilcoxon rank-sum test (non-parametric counterpart to the *t*-test) was used with the standard *α* value of 0.05 (Haynes, 2013). This test in particular was chosen as it is non-parametric in nature, and therefore does not make any assumptions regarding the distribution of the data. Because, when investigating aftereffects (discussed below), smaller sample sizes are used, assuming normally distributed data, as the *t*-test does, would not be appropriate.

The statistical significance of an aftereffect is determined in this study by comparing data from the observation phase to the baseline phase. The baseline phase data were treated as a static complete data set, while the data from the observation phase were tested incrementally. Since the observation phase is defined by its transience, one cannot simply test for significance between the baseline and observation phases in one pass. Therefore, the observation phase must be broken into small sections and tested in groups. After outlier detection was complete, the observation phase length was reduced from 600 to 576 gait cycles. The observation phase was then broken into 23 groups of 25 gait cycles, totaling 575 gait cycles. The last gait cycle (number 576) was simply ignored. The value of 25 was chosen for the group size as it allows for an adequate number of significance tests to be run, while still leaving a sufficient amount of data for each significance test. Each group of 25 gait cycles was then tested for significance against the entire baseline phase. Results of significance testing can be seen in the bottom right of each graph presented in this study, denoted by “**”. Where the line is present, statistical significance was found. Where the line is not present, no statistical significance was found. Since each of the 23 significance tests is done independently, no line, a solid line, or a “dashed” line can be present. Throughout the analysis, left-tailed, right-tailed, and two-tailed tests were used depending on the specific situation. The type of test that was used is conveyed on each graph above the significance line. An upward-facing arrow indicates that the test was performed against the alternative hypothesis of the observation phase being greater than the baseline phase (right-tailed). A downward-facing arrow indicates that the test was performed against the alternative hypothesis of the observation phase being less than the baseline phase (left-tailed). If no arrow is present, a two-tailed test was performed.

Lastly, for all graphs seen in this study, data were smoothed using second-degree polynomial local regression. This was done using a sliding window of 150 data points and was performed separately for each section of the experiment: baseline, adaptation, and observation. The only purpose of this smoothing is for a more clear visual representation. All significance testing (discussed above) used “unsmoothed” data.

## 3 Results

In an effort to show that aftereffects are a trend seen in the majority of subjects and not merely in a hand-picked subset, all data analyzed will be a composite of all 12 subjects tested. Therefore, all data is an average over the entire subject pool. Moreover, the main point of this study is to analyze the aftereffects produced by the perturbations in the adaptation phase. Consequently, we are not focusing on the data in the adaptation phase itself. That data will still, however, be presented in each figure, but will be greyed out in order to draw attention to the baseline and observation phases. Finally, all figures are color coded for added clarity. Blue represents the analysis of the left leg (perturbed side). Red represents the analysis of the right leg (unperturbed side). Purple represents either the analysis of the gait cycle as a whole or the analysis of asymmetry between legs. For asymmetric analysis, the right side parameter is always subtracted from the left.

### 3.1 Step length

This study shows that repeated unilateral stiffness perturbations on the Variable Stiffness Treadmill (VST) result in long-lasting aftereffects. Many of these aftereffects last for the full observation phase of the experiment (575 gait cycles) and appear to directly work toward correcting common issues seen in post-stroke gait dysfunction, like those discussed in Section 1. This will be examined further in Section 4.1.

**FIGURE 4:**
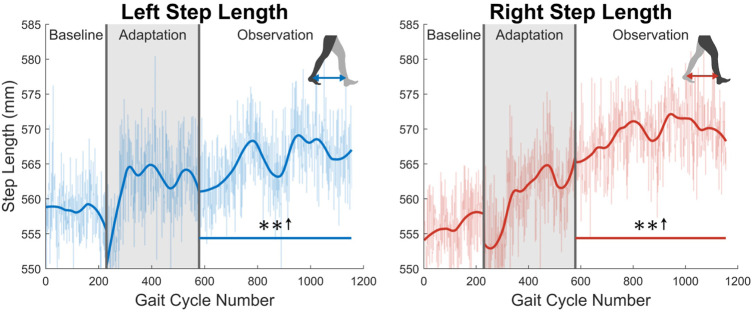
Left and right step length averaged for all 12 subjects. Step length was calculated as the projected distance between ankles in the floor plane at the time of heel strike. Both left and right step lengths are statistically significant for the entire observation phase, as indicated by the significance line. All significance testing was performed on “unsmoothed” data (seen in the lighter line). The darker line is the data smoothed by 2nd-degree polynomial local regression and was added only to allow the reader to more clearly see trends in the data.

**FIGURE 5:**
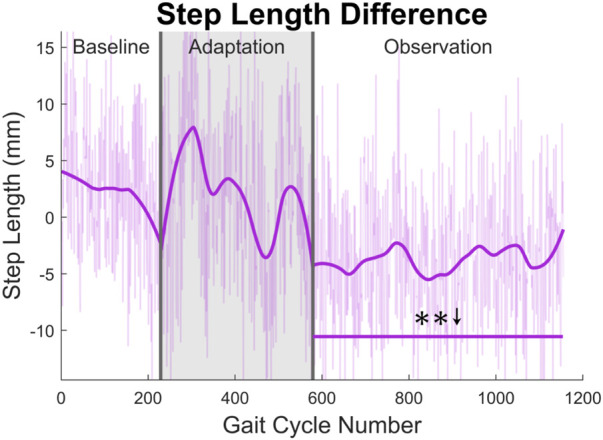
Step length asymmetry averaged for all 12 subjects. Step length was calculated as the projected distance between ankles in the floor plane at the time of heel strike. For this figure, step length asymmetry was found by subtracting right step length from left step length. The right step length is significantly greater for the entire observation phase, as indicated by the significance line. All significance testing was performed on “unsmoothed” data (seen in the lighter line). The darker line is the data smoothed by 2nd-degree polynomial local regression and was added only to allow the reader to more clearly see trends in the data.

At the highest level, meaningful aftereffects can be seen in terms of step length (see Figure 4). Step length in this study was measured as the Euclidean distance of the projection of the ankle markers of each leg onto the treadmill surface plane, at heel strike. For example, left step length is the distance between ankle markers at left heel strike, and *vice versa*. For the left leg, a statistically significant increase is seen when the observation phase is compared to the baseline phase, lasting for the entire observation phase. This increase has an average magnitude of 7.07 mm (1.27%). The right side is also significantly increased for the entire observation phase, but with an average magnitude of 13.07 mm (2.35%). It should be noted that, while the right leg is the unperturbed leg, it displays the larger step length increase. The asymmetry between left and right step lengths is also significant for the entire observation phase (see Figure 5).

### 3.2 Kinematics and kinetics

**FIGURE 6:**
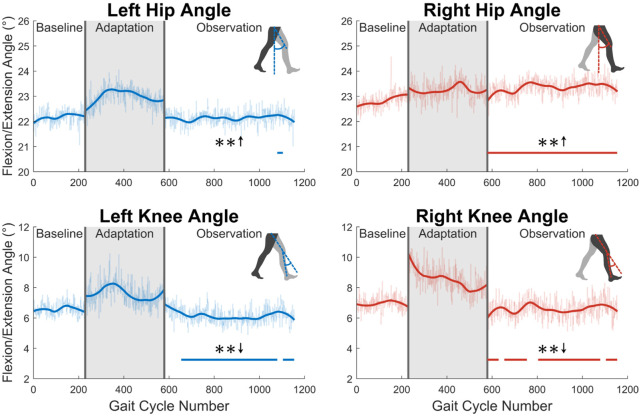
Hip and knee flexion/extension angles at heel strike for both left and right sides. Significance is shown in the observation phase as compared to the baseline phase by the significance line. All significance testing was performed on “unsmoothed” data (seen in the lighter line). The darker line is the data smoothed by 2nd-degree polynomial local regression and was added only to allow the reader to more clearly see trends in the data.

First, joint kinematics at heel strike will be examined. The left hip does not significantly increase its flexion angle at heel strike, as only one group of 23 was statistically significantly increased. The right hip flexion angle at heel strike however was significantly increased for the entire observation phase (see Figure 6). At the knee, a significant decrease in flexion is seen at heel strike for both left and right legs (see Figure 6). Both of these show statistically significant results for 83% of the observation phase.

**FIGURE 7:**
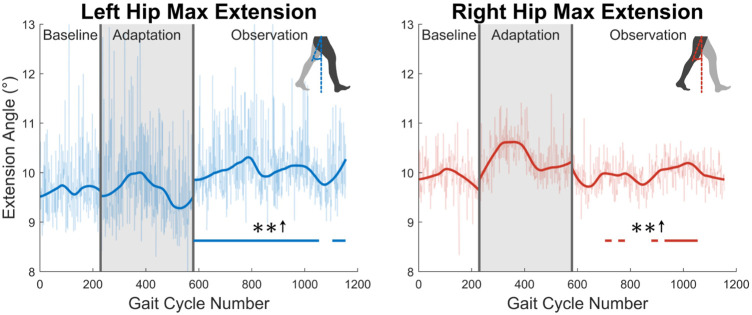
Maximum hip extension throughout the gait cycle for left and right legs. Note that values are positive because the extension angle was used instead of the flexion/extension angle for this figure. Significance is shown in the observation phase as compared to the baseline phase by the significance line. All significance testing was performed on “unsmoothed” data (seen in the lighter line). The darker line is the data smoothed by 2nd-degree polynomial local regression and was added only to allow the reader to more clearly see trends in the data.

Second, the trailing leg will be examined by considering the maximum hip extension angle created throughout the gait cycle (see Figure 7). The maximum extension angle (or minimum flexion-extension angle) made by the left leg is significant for nearly the entire observation phase (91%). The right leg however only shows significance for 35% of the observation phase.

**FIGURE 8:**
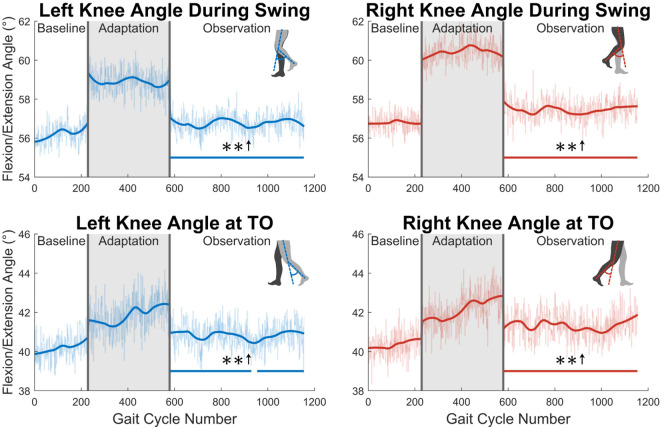
The top two graphs display maximum left and right knee flexion/extension angle during the swing phase. The bottom two graphs show knee flexion/extension angle at the instant that toe-off occurs. Significance is shown in the observation phase as compared to the baseline phase by the significance line. All significance testing was performed on “unsmoothed” data (seen in the lighter line). The darker line is the data smoothed by 2nd-degree polynomial local regression and was added only to allow the reader to more clearly see trends in the data.

Next, the knee joint is more closely examined. As shown in the top two graphs in Figure 8, subjects showed increased maximum knee flexion during the swing phase. This trend was statistically significant for the entire observation phase for both legs. Additionally, as shown in the bottom two graph in Figure 8, subjects increased their knee flexion angle at toe-off. This increase was statistically significant for the entire observation phase for the right leg, and nearly the entire observation phase for the left leg (96%).

**FIGURE 9:**
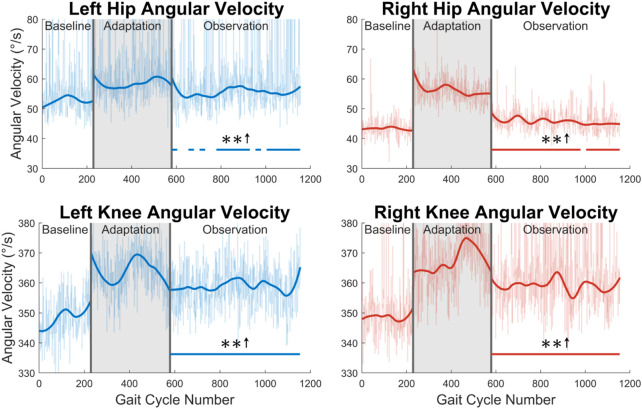
Maximum hip and knee angular velocities during the swing phase for both left and right legs. Significance is shown in the observation phase as compared to the baseline phase by the significance line. All significance testing was performed on “unsmoothed” data (seen in the lighter line). The darker line is the data smoothed by 2nd-degree polynomial local regression and was added only to allow the reader to more clearly see trends in the data.

Fourth, joint velocities during the swing phase were analyzed (see Figure 9). A significant increase in maximum flexion angular velocity is seen for a majority of the observation phase at both the left hip (70%) and the right hip (96%). For the knee, an increase in maximum extension angular velocity is seen for the entire observation phase for both legs (see Figure 9).

**FIGURE 10:**
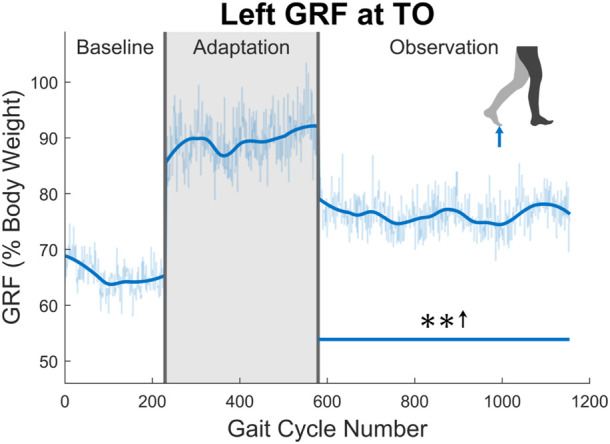
Maximum vertical ground reaction force between midstance and toe-off for the left leg in percent body weight. Significance is shown in the observation phase as compared to the baseline phase by the significance line. All significance testing was performed on “unsmoothed” data (seen in the lighter line). The darker line is the data smoothed by 2nd-degree polynomial local regression and was added only to allow the reader to more clearly see trends in the data.

Next, the ground reaction force (GRF) was examined. While the GRF was only available for the left leg due to hardware limitations, the trend of increased push-off force was observed for the entire observation phase (see Figure 10). The magnitude of this increase was quite notable at an average of 16.87% when comparing the observation phase to the baseline phase. This push-off force was defined as the second peak in the GRF curve during stance phase. The process ensured that push-off force was being analyzed and not heel strike force.

### 3.3 Muscle activity

In this section, muscle activity will only be discussed during the swing phase. This is where muscles can most easily be related to kinematics and step length, as will be discussed in Section 4.

**FIGURE 11:**
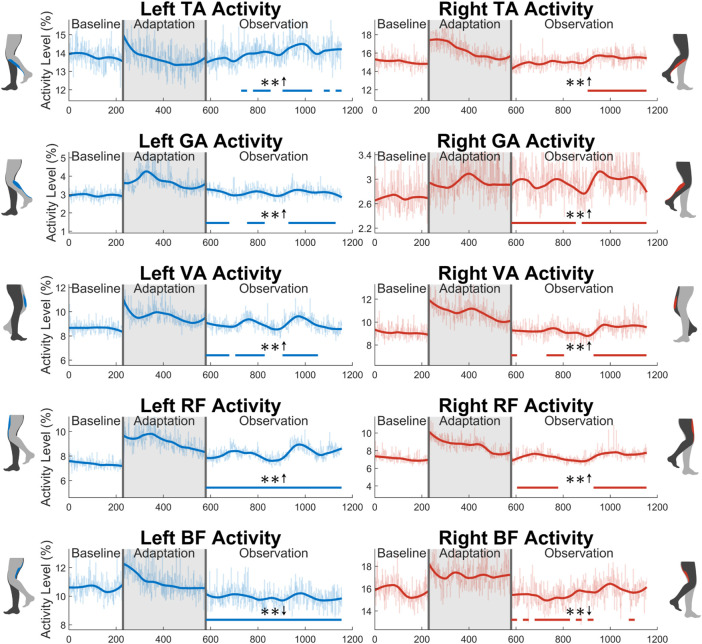
Average muscle activity during swing phase for all 10 muscles measured in this study: tibialis anterior, gastrocnemius, vastus medialis, rectus femoris, and biceps femoris. Significance is shown in the observation phase as compared to the baseline phase by the significance line. All significance testing was performed on “unsmoothed” data (seen in the lighter line). The darker line is the data smoothed by 2nd-degree polynomial local regression and was added only to allow the reader to more clearly see trends in the data.

The average activity level during the swing phase of all 10 muscles measured can be seen in Figure 11. Starting with the tibialis anterior (TA), significance can only be seen for part of the observation phase for both the left leg (48%) and the right leg (43%). Next, the gastrocnemius (GA) shows a statistically significant increase in the left leg for 65% of the observation phase. A significant increase is seen for 22 out of 23 (96%) of the observation groups for the right leg. For the vastus medialis (VA), significance is seen for part of the observation phase for both the left (65%) and right (57%) sides. Next, a significant increase is seen in the left rectus femoris (RF) for the entire observation phase. This can be observed to a lesser degree on the right side, as only 70% of the groups were statistically significant. Similarly, a reduction in biceps femoris (BF) activation is seen more clearly on the left side (100%) than on the right side (48%).

### 3.4 Gait cycle timing

**FIGURE 12:**
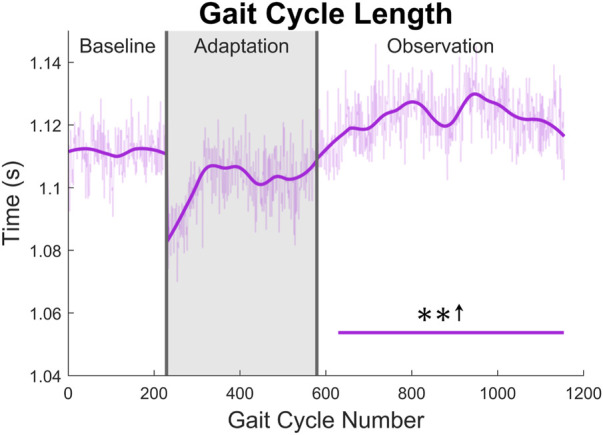
Gait cycle length with respect to time (measured from left heel strike to left heel strike). Significance is shown in the observation phase as compared to the baseline phase by the significance line. All significance testing was performed on “unsmoothed” data (seen in the lighter line). The darker line is the data smoothed by 2nd-degree polynomial local regression and was added only to allow the reader to more clearly see trends in the data.

The gait cycle can also be analyzed temporally. First, and most simply, gait cycle length (with respect to time), can be measured by finding the elapsed time between left heel strikes. A significant increase can be seen for the entire observation phase (see Figure 12).

**FIGURE 13:**
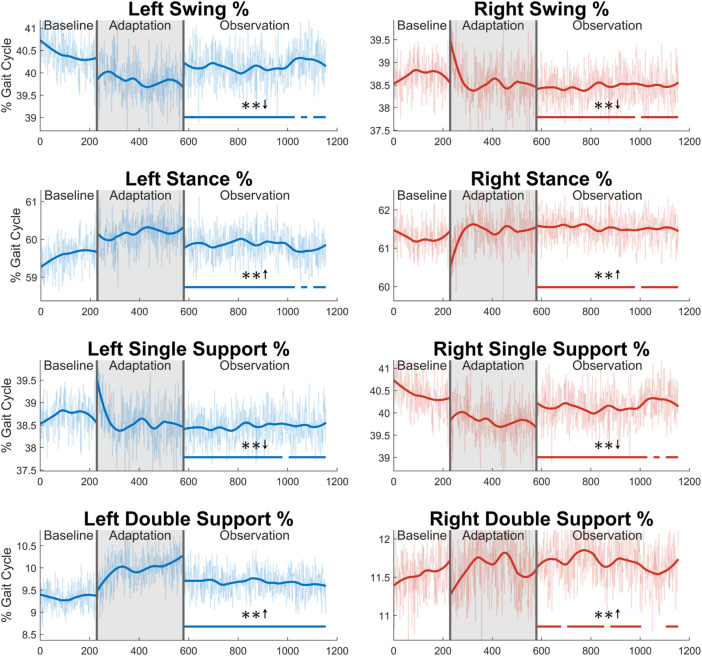
Sections of the gait cycle displayed with respect to how much of the entire gait cycle they occupy (in terms of percentage). The double support phase is determined to be left or right depending on which foot is in front and has more recently achieved heel strike. Significance is shown in the observation phase as compared to the baseline phase by the significance line. All significance testing was performed on “unsmoothed” data (seen in the lighter line). The darker line is the data smoothed by 2nd-degree polynomial local regression and was added only to allow the reader to more clearly see trends in the data.

Next, the gait cycle can be more deeply analyzed by examining how long (in terms of percentage) the subjects spent in each section of the gait cycle (see Figure 13). First, for swing phase, a significant decrease for a majority of the observation phase can be seen in both the left side (91%) and the right side (96%). As expected, the exact opposite trend is seen in the stance phase for both legs. Next, significant decreases can be seen in the left single support phase (96%) and right single support phase (91%). Predictably, the left and right double support phases show the opposite trend with similar levels of significance (100% for the left side, 74% for the right side). While it is not displayed graphically, the same analysis was performed in terms of time instead of percentage. The results for stance and double support were nearly identical to those seen in Figure 13, and the results for swing and single support were largely insignificant.

**FIGURE 14:**
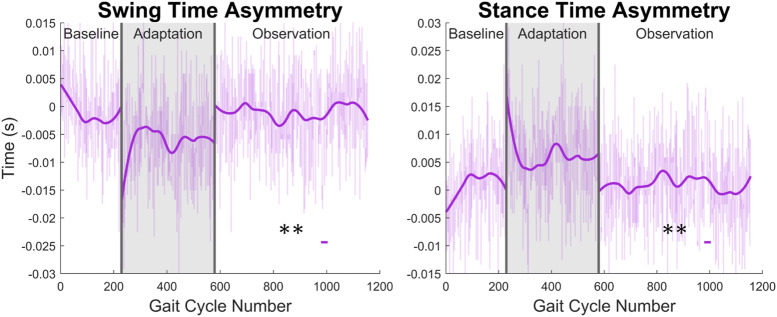
Swing and stance time asymmetry in terms of time. For the swing phase, asymmetry was found by subtracting the time spent in right swing from the time spent in left swing. For the stance phase, the same process was used. Significance is shown in the observation phase as compared to the baseline phase by the significance line. All significance testing was performed on “unsmoothed” data (seen in the lighter line). The darker line is the data smoothed by 2nd-degree polynomial local regression and was added only to allow the reader to more clearly see trends in the data.

From Figure 13 and the discussion above, it would appear that there is not any significant asymmetry in terms of gait cycle timing. This can be further confirmed by investigating more directly the asymmetry with respect to swing and stance (see Figure 14). Swing time asymmetry was calculated for each gait cycle by subtracting the time the right leg spent in the swing phase from the time the left leg spent in the swing phase. For stance time asymmetry, the same method was used. Significance is only found in 1 out of 23 groups (4%) for both swing time asymmetry and stance time asymmetry.

## 4 Discussion

The results of this study suggest that the Variable Stiffness Treadmill (VST), *via* its unilateral low stiffness perturbations, could be a very useful tool with respect to post-stroke gait rehabilitation. This section will dive further into stroke rehabilitation, mention the shortcomings of this study, and contemplate future applications of the results presented in this paper.

### 4.1 Stroke rehabilitation

The VST shows the potential to be instrumental in post-stroke gait rehabilitation, as it produces long-lasting aftereffects after a single 10-min intervention. The asymmetric environment this device creates appears to engage subjects in a unique way. It is important to note that many of the aftereffects analyzed last for the entire observation phase of the experiment (575 gait cycles). With only 400 perturbed gait cycles, the length of this aftereffect is quite substantial, and to our knowledge has not been shown before. Additionally, the aftereffects for many parameters analyzed (step length included) only ended due to the length of the experiment. While we currently do not know the true duration of these aftereffects, it is possible that they continue for many more gait cycles beyond the duration of this study.

**FIGURE 15:**
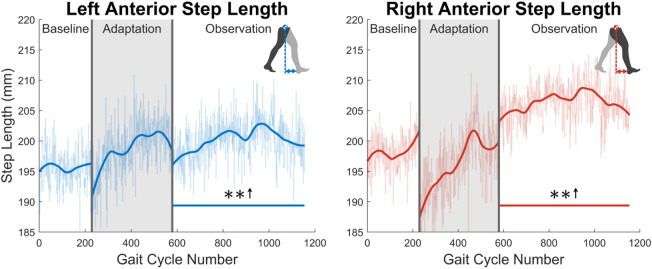
Left and right anterior step length averaged for all 12 subjects. Anterior step length was calculated as the anterior/posterior distance between the center of mass and the leading heel at heel strike. Significance is shown in the observation phase as compared to the baseline phase by the significance line. All significance testing was performed on “unsmoothed” data (seen in the lighter line). The darker line is the data smoothed by 2nd-degree polynomial local regression and was added only to allow the reader to more clearly see trends in the data.

As was discussed in Section 1, post-stroke gait is often characterized first by step length asymmetry and an overall decrease in step length on both sides (Titianova et al., 2003; Nadeau, 2014). The aftereffect of asymmetrical step length increase seen in this study directly counteracts said issue. The argument can be made that because step length is measured from ankle to ankle, it is dependent on the trailing leg as much as the leading leg. Therefore, this increase in step length could simply be caused by each subject’s trailing leg “riding” the treadmill for longer. While this theoretically could be the case, this theory can be disproven by investigating step length in a different fashion or looking at a different variable. Analyzing the distance from the leading foot to the center of mass, which we’ll call anterior step length, shows that subjects are in fact placing their leading foot farther in front of their center of mass (see Figure 15). A significant increase in anterior step length is seen for the entire observation phase. More explicitly, anterior step length is measured as the anterior/posterior distance between the center of mass and the leading heel at heel strike. As only the lower body was tracked during this study, the center of mass is estimated as the average position of the following four markers around the hips: left anterior superior iliac, right anterior superior iliac, left posterior superior iliac, and right posterior superior iliac (see Figure 3). This analysis suggests that the step length aftereffects produced by unilateral stiffness perturbations on the VST are in part caused by swinging the leg farther forward prior to heel strike. This appears to relate to the behavior seen in subjects post-stroke (Hirata et al., 2019).

While step length and step length asymmetry characterize post-stroke gait at the broadest level, kinematics can help explain the cause of such gait and how it can be corrected. The kinematic results analyzed in Section 3.2 both support and help explain the observed step length aftereffects as well as address more specific kinematic issues commonly found in post-stroke gait as discussed in Section 1. We can see that this step length increase is explained kinematically first by increased hip flexion. Reduced hip flexion during the swing phase is a quite common behavior post-stroke and may be a major reason for the asymmetric step length that is often seen (Balaban and Tok, 2014). The results of this study seem to directly counteract this behavior. Recalling that the right leg produced the larger step length during the observation phase, this behavior can at least be explained in part by the right hip having an increased level of flexion when the right heel made initial contact. While for the hip, more flexion at heel strike helps produces a larger step length, more extension is required at the knee to assist in increasing step length. Since there is less flexion at the knee joint, the foot can be placed farther in front of the subject’s center of mass at heel strike, again aiding in explaining how an increase in step length is achieved. Another common post-stroke trend is reduced hip extension during the stance phase when approaching push-off (Balaban and Tok, 2014). Not only does this reduce overall step length, but also limits the amount of forward propulsion that can be generated during terminal stance. The aftereffect of increased max hip extension during stance seen in this study again appears to directly work toward correcting common post-stroke gait dysfunction behavior.

Looking more closely at the knee joint, an aftereffect of higher maximum flexion during swing is seen. For a larger step length to be achieved, the leg must travel more distance during the swing phase. One way that humans maximize step length while keeping energy expenditure low is by reducing the moment of inertia of the swing leg. This is most commonly achieved by flexing the knee more during the swing phase (Smith and Hanley, 2013). Additionally, this higher knee flexion during swing appears to directly counteract the common post-stroke trend of more knee extension during the swing phase (Balaban and Tok, 2014). This post-stroke trend is thought to be at least partially responsible for foot drop (or toe drag), which is known to be a major cause of falling (Little et al., 2014; Matsuda et al., 2017). The aftereffect of increased knee flexion could be beneficial in creating enough clearance between the ground and the swing foot to avoid tripping. Next, an aftereffect of increased knee flexion at toe-off was observed for both left and right legs. The reason for this behavior is not quite as clear. It is possible that subjects were simply preparing for the increased maximum flexion angle that was about to be achieved during the ensuing swing phase. It is also feasible that increased knee flexion at toe-off indicates an early transfer of weight to the front leg in preparation for faster walking or increased step lengths. This trend is meaningful though, as reduced knee flexion is a common behavior in post-stroke gait (Chen et al., 2005). While the exact connection between increased knee flexion at toe-off and larger step length is not known for certain, this behavior appears to be promising in terms of post-stroke gait rehabilitation. In terms of kinematics, the last parameters to discuss are the angular velocities at the hip and knee joints during the swing phase. While, to our knowledge, these have not been investigated in stroke subjects to the same degree as the other parameters discussed, simply considering the dynamics of the swing leg can provide insight into why joint velocities are so important. First, another mode of generating a larger step length is increasing the momentum of the swing leg. Simply put, if the leg is moving at a higher speed during swing phase, a larger step can more easily be achieved. Examining the maximum angular velocity of the hip and knee during swing gives insight into this idea. Both an increase in hip flexion speed and knee extension speed add momentum to the swing leg and allow it to be swung farther forward prior to heel strike. Also, since increased walking speed is a major goal of post-stroke gait rehabilitation (Patterson et al., 2008, 2010), consider how joint speed impacts walking speed. Flexing the hip and extending the knee at a faster rate during swing allows for gait speed to be increased through two distinct modes: increasing step frequency while holding step length constant or increasing step length while holding step frequency constant. While these other parameters (increased step length and increased step frequency) *may* be accompanied by an increased walking speed, they are not as directly related to walking speed as joint speed is.

Aside from kinematics, the ground reaction forces help explain the larger step lengths and again seem to work toward correcting the common post-stroke issue of reduced propulsion (Chen et al., 2005). Simply put, pushing off the ground with more force will allow the leg to be swung faster and farther forward, resulting in a larger step length. It is important to note that the ground reaction forces captured in this study are solely in the vertical direction. While this is not the total propulsion force that is presented in other gait studies, the vertical force is related to the total force through simple geometry. Interestingly enough, the larger vertical forces were seen when a larger amount of hip extension was taking place in terminal stance. Assuming that, as hip extension increases, the push-off force becomes more in the horizontal direction, one would think that the vertical ground reaction force read on the VST would decrease. The fact that this force actually increases, suggests that the true propulsion force aftereffect is significantly greater than what is presented in this paper.

Concerning muscle activity, many of the muscles observed in this study help explain the increased step length, as was discussed in Section 3.3. The TA in particular is of great importance for post-stroke gait rehabilitation. One of the most common issues in post-stroke gait is reduced dorsiflexion during swing (Balaban and Tok, 2014). This behavior can lead to toe drag (Von Schroeder et al., 1995), which is one of the most common modes of falling in stroke victims. A slight aftereffect of increased TA activation was seen in this study. An increase in TA activity during the swing phase could be one factor that allows for more ankle dorsiflexion, increasing the clearance level between the foot and ground Intiso et al. (1994); Westhout et al. (2007). Even though an increase in TA activity was seen, it was not accompanied by an increase in dorsiflexion in the healthy subjects who participated in this study. It is feasible that, due to joint limitations, a significant increase in dorsiflexion cannot be achieved in individuals who already dorsiflex a healthy amount, but this is merely speculation. This will need to be further tested with more healthy individuals and stroke victims to be able to discuss this topic with more confidence. Moving past the TA, the GA assists in knee flexion. It is presumed that the increased activation seen helps explain the changes in knee flexion discussed in Section 3.2 to a degree. Next, the increase in VA activity can help describe the increase in knee extension speed also discussed in Section 3.2. For the RF, an increase in activity can assist in explaining the increase in hip flexion that helped generate the increase in step length. Next, a reduction in BF activation can assist in explaining the increased hip flexion discussed in Section 3.2. Noting that the BF is a hip extensor, a decrease in its activity may allow for more hip flexion *via* the hip flexors, such as the RF. It is difficult to identify further specific common post-stroke gait muscle activity behaviors to relate to the concepts just discussed. While dysfunction in muscle activity is certainly present post-stroke, specific behaviors vary considerably from subject to subject (Den Otter et al., 2007; Daly et al., 2010). In general, a reduction of overall muscle activity is commonly seen with stroke subjects, especially on the paretic side of their body (Olney and Richards, 1996; Chen et al., 2005). While only a small set of muscles were analyzed in this study, a general aftereffect trend of increased muscle activity is seen. On both the left and right sides, 80% of the observed muscles were significantly increased for at least half of the observation phase. While an experiment measuring more muscles would need to be performed to say this more confidently, the results presented here are at a minimum trending in the correct direction.

Concerning gait cycle timing, it most likely does not help to understand function changes, such as increased step length, but is more likely a result of such changes. This can be seen clearly when examining gait cycle length with respect to time. The increase seen appears to be directly linked to the step length increase observed. As subjects are walking at a fixed speed on the treadmill, for larger steps to be taken, the gait cycle needs to be accomplished over a longer period. Otherwise, the subject would begin to walk faster than the treadmill belts are moving. Gait cycle timing parameters do, however, help characterize and differentiate between healthy and post-stroke gait. Additionally, the gait cycle timing data presented in this study only appears to work toward correcting common post-stroke gait issues to a small degree. While a few specific behaviors, such as a prolonged swing phase (Titianova et al., 2003; Chen et al., 2005; Nadeau, 2014) and decreased double support time (Nadeau, 2014), do appear to be corrected by our findings, many other issues are not properly addressed. It is possible that these results could improve with subjects whose gait is already asymmetric, but this would require future investigation. These limitations will be further discussed in the next section.

While there are many significant aftereffects presented in this study, we must question whether or not they are useful to stroke rehabilitation. It has been debated whether or not these short-term aftereffects in healthy subjects are genuine indicators of the possibility of long-term, neuroplastic, functional changes in stroke subjects (Reinkensmeyer and Patton, 2009; Huang et al., 2011). First, when comparing stroke subjects with healthy subjects, multiple studies have shown that asymmetric gait training translates to both populations in a similar fashion after a single therapy session (Reisman et al., 2005, 2007, 2009). This comparison has been previously investigated using age-matched and gender-matched healthy control subjects. One study even notes a more robust aftereffect in the post-stroke subjects than in the healthy subjects (Reisman et al., 2009). Second, the topic of creating long-lasting effects must be considered. While this topic has yet to be thoroughly explored, one study suggests that transient aftereffects can be capitalized on through a regimented training program (Reisman et al., 2013). This study showed that through repeated error augmentation therapy sessions with stroke patients, trends of step length asymmetry improvements were evident at both 1-month and 3-month check-ins periods (Reisman et al., 2013). While this environment of unmatched belt speeds differs from the unilateral stiffness perturbations performed in our study, both environments are asymmetric in their nature and resulting functional changes. We are hoping that these seemingly useful aftereffects can be effective in creating long-term corrective outcomes. As mentioned in Section 4.3, this protocol will first need to be tested with stroke patients through a single therapy session, and then eventually through a repetitive training program.

### 4.2 Shortcomings

While the data presented in this study is indeed quite promising, the study did have its limitations. Regarding the experimental design, first, the observation phase of the experiment is not long enough to capture many of the aftereffects in their entirety. While this is in one sense good news, because long-lasting aftereffects are the goal, having a fuller understanding of the duration would be beneficial. Next, only having access to ground reaction forces on one side of the treadmill leaves many questions unanswered. The results obtained from the left side were quite encouraging, but having access to both sides would allow for a deeper analysis and understanding of human gait in this environment. Finally, while this experiment was not designed to explain different stiffness levels, simply treating 45 kN/m as “low stiffness” and 1 MN/m as “high stiffness” raises many questions in terms of stiffness level. At this point, we do not have a good understanding of how different stiffness levels would affect human gait aftereffects.

With respect to the results of the experiment, most of the shortcomings are related to gait cycle timing (discussed in Section 3.4), as this is what many of the common post-stroke behaviors discussed in Section 1 refer to. Several of these behaviors were either not improved by the results presented, or the results work in a counteractive way. Some examples of these trends seen in stroke patients are the following: prolonged swing phase (Titianova et al., 2003; Chen et al., 2005; Nadeau, 2014), reduced single support time (Chen et al., 2005), and increased double support time (Nadeau, 2014) for the affected leg, and prolonged stance phase (Olney and Richards, 1996) and decreased swing time (Nadeau, 2014) for the unaffected leg. While we believe that the results, as a whole, presented in this paper greatly outweigh the shortcomings addressed here, they are important to note for future study and experiment design.

### 4.3 Implications and future applications

This study is quite encouraging in the field of robot-assisted post-stroke gait rehabilitation. The aftereffects found after a single walking session are unmatched in the literature according to our knowledge. We hope that this study helps lead to patient-specific repeated interventions that treat each stroke subject based on their individual needs.

We hope to accomplish this by first gaining a fuller understanding of the perturbations created on the VST. This could be accomplished first by performing similar experiments with varying stiffness levels and section duration. Additionally, having a smaller subset of subjects come back regularly for VST interventions could help us understand the longer-term effects of repeated unilateral stiffness perturbations and how they could be used in a clinical setting. Another great future step would be to test the same, or a similar protocol, with a subject pool of stroke patients. While the results are promising with healthy subjects, we hope to soon reproduce these results with stroke patients.

Finally, and possibly most importantly, we believe that modeling this behavior is an integral part of the process of creating an effective post-stroke gait rehabilitation protocol. As was discussed in Section 4.1, the issues presented with stroke are unique to every stroke case. Therefore, to have a truly robust rehabilitation process, we must not “paint with broad strokes,” but be able to meet each subject’s specific needs. Such a complex issue requires a robust model that can simulate an individual’s behaviors and dysfunction and then solve for the best possible mode of intervention. While progress is being made in this area (Chambers and Artemiadis, 2021), further research and more studies are required.

Currently, the results presented and discussed in this paper show much promise toward achieving the future goal of a robust, post-stroke gait rehabilitation protocol. This study suggests that the unilateral stiffness perturbations created on the Variable Stiffness Treadmill may be able to assist in correcting many of the problems generally seen in gait post-stroke. We show a significant, asymmetric increase in step length that lasts at least 575 gait cycles, which is supported by kinematics, kinetics, muscle activity, and gait timing data. Based on these results, we believe the main contribution of this paper is a deeper analysis of a promising therapy protocol that is achieved using our unique robotic treadmill. We hope that extensions of this study will drastically improve the landscape of post-stroke gait rehabilitation in the future.

## Data Availability

The raw data supporting the conclusion of this article will be made available by the authors, without undue reservation.
