# Supplementary material for: Using robot-assisted stiffness perturbations to evoke aftereffects useful to post-stroke gait rehabilitation
Source: Front Robot AI. 2023 Jan 4;9:1073746. doi: 10.3389/frobt.2022.1073746 (PMC9846529; doi:10.3389/frobt.2022.1073746)
Supplement: Supplementary file 1 [file DataSheet1.PDF]

Supplementary Material

1 SUPPLEMENTARY TABLES AND FIGURES

1.1 Tables

Table S1. Left Step Length Correlation Analysis with respect to Kinematics, Muscle Activity, and Kinetics during the Observation Phase.

	**Left Step Length**	
	Correlation coefficient	p-value
Left Anterior Step Length	0.604	**0.000**
Left Hip Angle at Heel Strike	0.232	**0.000**
Left Knee Angle at Heel Strike	-0.263	**0.000**
Max Left Hip Extension	0.063	0.132
Max Left Hip Swing Velocity	0.035	0.400
Max Left Knee Swing Velocity	-0.038	0.368
Left TA During Swing	0.191	**0.000**
Left GA During Swing	-0.004	0.928
Left VA During Swing	0.134	**0.001**
Left RF During Swing	0.192	**0.000**
Left BF During Swing	-0.134	**0.001**
Left Push-Off Force	-0.185	**0.000**

Table S2. Right Step Length Correlation Analysis with respect to Kinematics and Muscle Activity during the Observation Phase.

	**Right Step Length**	
	Correlation coefficient	p-value
Right Anterior Step Length	0.239	**0.000**
Right Hip Angle at Heel Strike	0.281	**0.000**
Right Knee Angle at Heel Strike	-0.135	**0.001**
Max Right Hip Extension	0.063	**0.000**
Max Right Hip Swing Velocity	-0.153	**0.000**
Max Right Knee Swing Velocity	0.044	0.109
Right TA During Swing	0.302	**0.000**
Right GA During Swing	0.057	0.173
Right VA During Swing	0.122	**0.003**
Right RF During Swing	0.165	**0.000**
Right BF During Swing	0.003	0.947

1.2 Figures

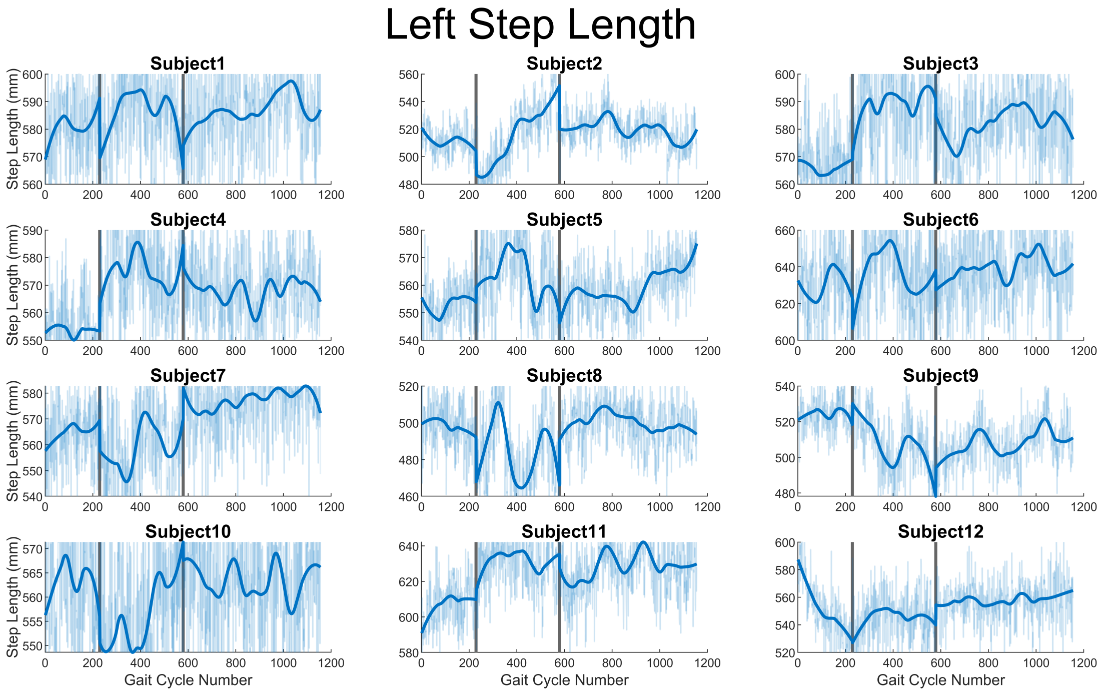

Figure S1. Left step length for all 12 subjects. Step length was calculated as the projected distance between ankles in the floor plane at the time of heel strike. The darker line is the data smoothed by 2nd-degree polynomial local regression and was added to allow the reader to more clearly see trends in the data.

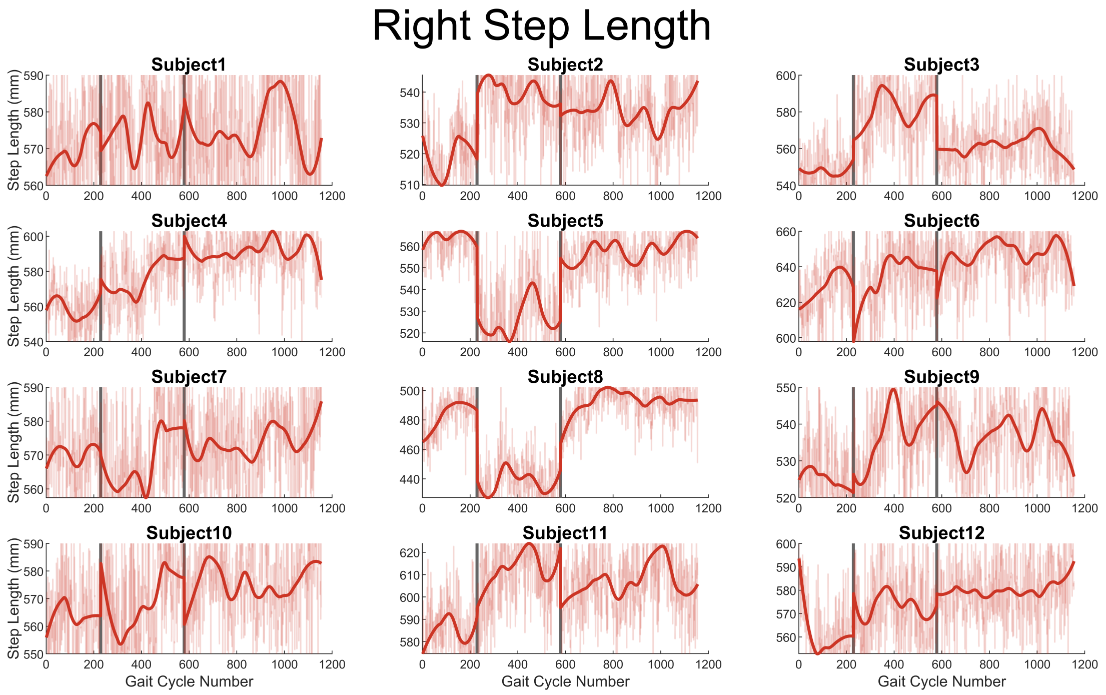

Figure S2. Right step length for all 12 subjects. Step length was calculated as the projected distance between ankles in the floor plane at the time of heel strike. The darker line is the data smoothed by 2nd-degree polynomial local regression and was added to allow the reader to more clearly see trends in the data.

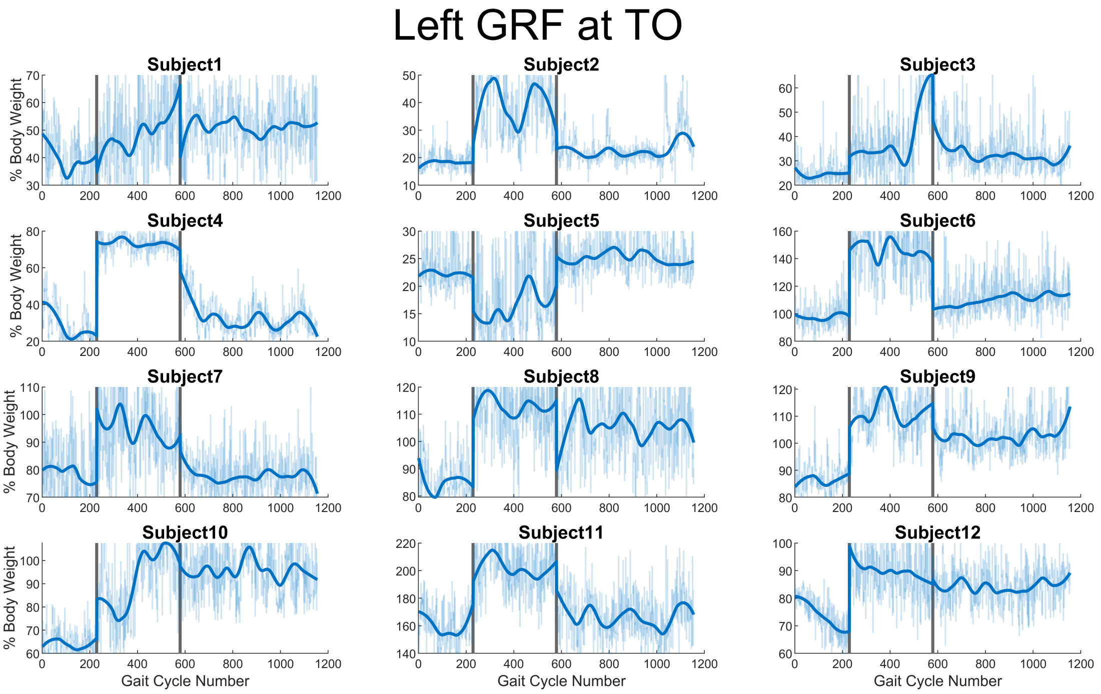

Figure S3. Maximum vertical ground reaction force between midstance and toe-off for the left leg in percent body weight for all 12 subjects. The darker line is the data smoothed by 2nd-degree polynomial local regression and was added only to allow the reader to more clearly see trends in the data.

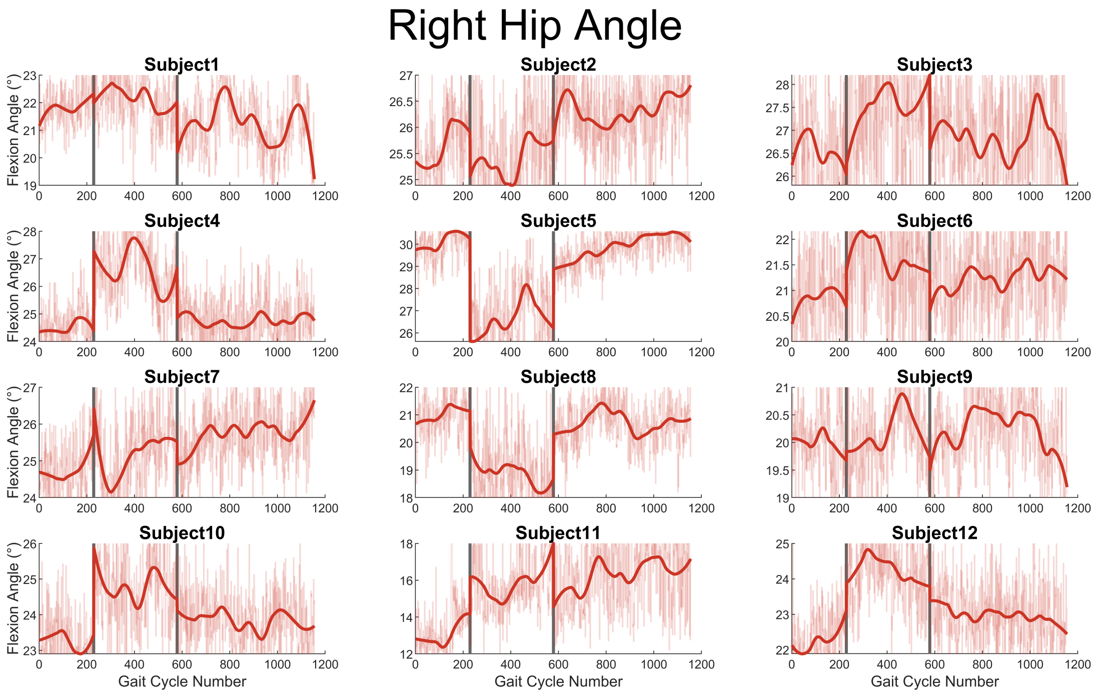

Figure S4. Right hip flexion/extension angles at heel strike for all 12 subjects. The darker line is the data smoothed by 2nd-degree polynomial local regression and was added only to allow the reader to more clearly see trends in the data.

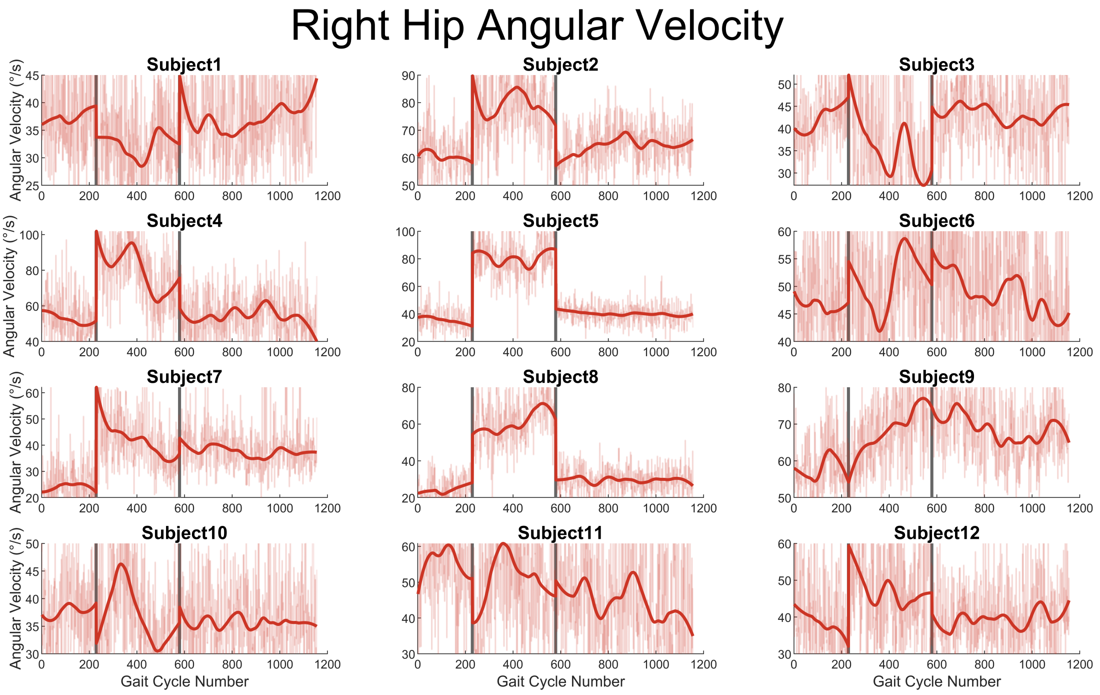

Figure S5. Maximum right hip angular velocities during the swing phase for all 12 subjects. The darker line is the data smoothed by 2nd-degree polynomial local regression and was added only to allow the reader to more clearly see trends in the data.

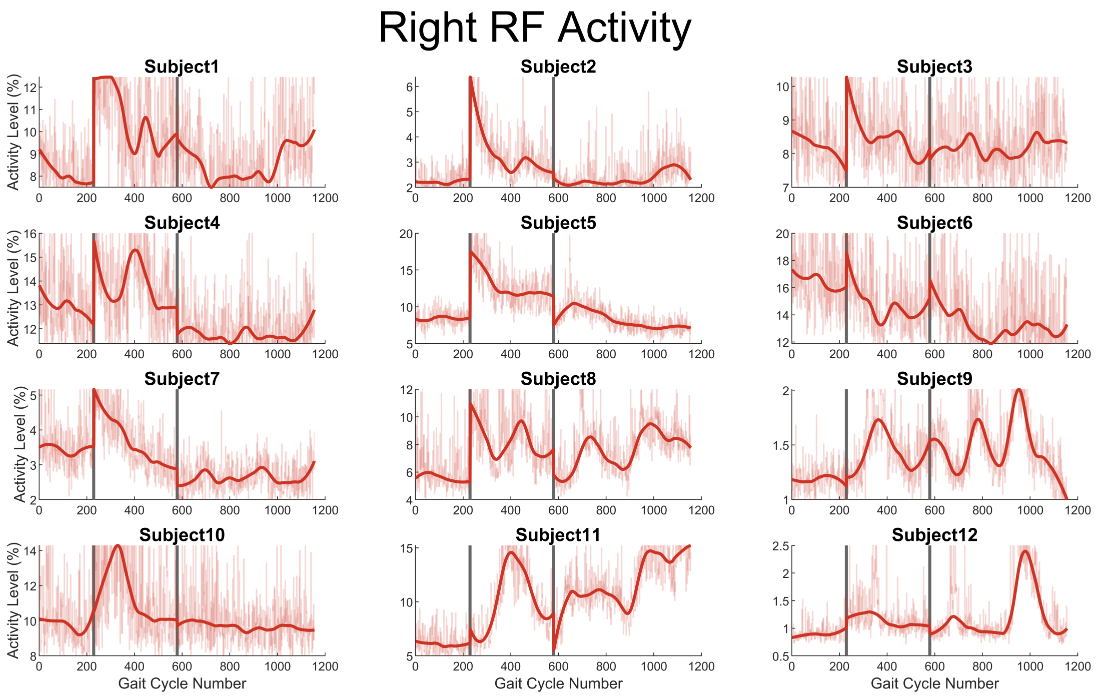

Figure S6. Rectus femoris (RF) muscle activity data averaged over swing phase for all 12 subjects. The darker line is the data smoothed by 2nd-degree polynomial local regression and was added only to allow the reader to more clearly see trends in the data.
